# Seeking snow and breathing hard – Behavioral tactics in high elevation mammals to combat warming temperatures

**DOI:** 10.1371/journal.pone.0225456

**Published:** 2019-12-11

**Authors:** Wesley Sarmento, Mark Biel, Joel Berger

**Affiliations:** 1 Wildlife Biology Program, The University of Montana, Missoula, Montana, United States of America; 2 Glacier National Park, West Glacier, Montana, United States of America; 3 Department of Fish, Wildlife, and Conservation Biology, Colorado State University, Fort Collins, Colorado, United States of America; 4 Wildlife Conservation Society, Bronx, New York, United States of America; Wildlife Conservation Society Canada, CANADA

## Abstract

The world glaciers and areas of persistent summer snowpack are being lost due to warming temperatures. For cold-adapted species, habitat features may offer opportunities for cooling during summer heat yet the loss of snow and ice may compromise derived thermoregulatory benefits. Herein we offer insights about habitat selection for snow and the extent to which other behavioral adjustments reduce thermal debt among high elevation mammals. Specifically, we concentrate on respiration in mountain goats (*Oreamnos americanus*), a species whose native distribution is currently tied to northern mountain ranges of North America, where large patches of persistent summer snow are declining, and which became extinct during geologically warmer epochs. To examine sensitivity to possible thermal stressors and use of summer snow cover, we tracked marked and unmarked mountain goats in Glacier National Park, Montana, USA, to test hypotheses about selection for cold microclimates including shade and snow during periods of relatively high temperature. To understand functional responses of habitat choices, we measured microhabitat temperatures and a component of goat physiology–breaths per minute–as an index for metabolic expenditure. Individuals 1) selected areas closer to snow on warmer summer days, and 2) on snow had a 15% mean reduction in respiration when accounting for other factors, which suggests remnant snow plays an important role in mediating effects of air temperature. The use of shade was not as an important variable in models explaining respiration. Despite the loss of 85% of glaciers in in Glacier National Park, summer’s remnant snow patches are an important reservoir by which animals reduce heat stress and potential hyperthermia. Our findings, when contextualized with behavioral strategies deployed by other high elevation mammalian taxa help frame how ambient temperatures may be modulated, and they offer a direct way by which to assess susceptibility to increasing heat in cold-adapted species.

## Introduction

Global climate change is altering ecological systems through long-term changes in weather patterns, particularly temperature, precipitation, and glacial loss [1]. The alterations are more pronounced closer to the poles and in higher elevations [2], sites where water is frequently isolated year-round in glaciers or snowpack. Warming temperatures are reducing these hydrologic features [3], and simultaneously altering microclimates [2] which cold-adapted species maintain thermal reliance [4]. These hydrologic and geomorphic alternations will subsequently affect ecological interactions and species distributions, often mechanistically by physiological impacts on individuals.

Mammalian taxa use different and often well-known proximate behaviors to optimize their metabolism when directly challenged with weather-related change. To generate warmth for instance some primates huddle; among rodents and lagomorphs, marmots, pikas, and African ice rats maximize solar radiation on brisk days (Table 1). Cold-adapted species of boreal, montane, or tundra-restricted distributions face different challenges and are likely to be impacted by decreasing snowpack, a symptom of increasing global temperature (Table 1).

**Table 1:** Behaviors to modulate heat gain or loss at high elevation* in select mammal orders.

Order	Common Name	Latin	Primary Locale	Reducing Heat Load	Increasing Heat Load
**Primates**					
	Black Snub-nosed monkey	*Rhinopithecus spp*.	Mountains of Yunnan, China	?	huddle; use south facing and sunnier slopes[5]
**Rodentia**					
	Marmot	*Marmota flaviventris*	Rocky Mts, USA	burrow use	basking (solar radiation), social hibernation[6]
	African Ice Rat	*Otomys sloggetti *	Drakensberg Mts, SA		social basking[7]
**Lagomorphs**					
	Pika	*Ochotona princeps*	Glacier Park—Rocky Mts USA	rock crevasses, north-facing slopes	basking (solar radiation)[8–10]
**Ungulata**					
	Mountain Goats	*Oreamnos american*	Glacier Park—Rocky Mts USA	resting on snow, panting ^a^	?
	Muskoxen	*Ovibos moschatus*	western Arctic Alaska	resting on snow; standing on windy ridges[11,12]	?
	Moose	*Alces*	boreal Alberta, Canada	shade seeking; open-mouth panting[13]	?
	Wild Yaks	*Bos mutus*	Tibetan Plateau	standing in water, use of windy ridges[14,15]	?

*Moose are primarily a boreal species although they occur in tundra ecosystems that reach to the Arctic Ocean.

^a^ This study.

Across the Rocky Mountains of the USA, the persistence of summer snow cover is predicted to shrink by 63% by 2099 and will impact many species. Reduction in summer snow is leading to range contraction for the wolverine (*Gulo gulo*)[16], a species that uses snowpack to preserve winter-killed ungulates [17]. Reliance on sympagic environments–those with water bound as ice–is becoming increasingly clear for some cold-adapted mammals. And, as water is unbound from ice during winter, effects can be negative. Caribou (*Rangifer tarandus*) are vulnerable to rain-on-snow events [18], and the young of muskoxen (*Ovibos moschatus*) exposed to rain-on-snow when in-utero experience retardation in later skeletal growth [11]. And, the sublimation of winter snow on the Tibetan Plateau reduces broad swaths of habitat for endangered wild yaks (*Bos mutus*), a particular alarming situation because snow is required as a substitute for water during winter and requisite to support costs of lactation during winter [15,19]. Nevertheless, for most cold weather specialists inhabiting peri-glacial zones, little remains known about if, or how, immediate behaviors are deployed when challenged by warming temperatures.

Respiration is one such response, a means by which mammals may modulate body heat [20] and avoid effects of hyperthermia [13,21], especially for species with thick pelages and/or poorly developed sweat glands [22]. Respiratory thermoregulation, however, is limited because it reduces body temperatures slowly [23,24], and excises metabolic costs including increased oxygen demand, energy expenditure, and the loss of water/electrolytes [22].

Herein we address the knowledge gap on proximate behavioral strategies that a cold adapted species uses to mitigate against warming temperatures in a previously glaciated ecosystem with rapid losses of ice and snow. We assess one primary level of habitat selection–snow patches–and then assess the extent to which ambient temperatures associated with snow modulates respiration. Our surrogate measure is panting, a metric rarely evaluated in putatively climate-sensitive species of tundra or alpine zones in part because observations are frequently difficult and especially for organisms whose biology is inextricably linked to mountain precipices [25,26]. We concentrate on mountain goats (*Oreamnos americanus*), and test the more general hypothesis that snow patch usage reduces summer heat load. A number of predictions logically follow. More specifically, that: a) associations between individuals and snow patches are non-random when temperature is taken into account; b) resting on snow reduces respiration; and c) snow patches receive relatively higher usage than ridges on windy days, this latter prediction intended to match thermal balance against the competing hypothesis that insect avoidance plays a larger role in habitat selection.

Our rationale concerning thermal determinants of goat distribution in alpine and sub-alpine environments is predicated on the observation that the continental distribution of mountain goats has receded north and coincides with local extinctions along a southern periphery as Holocene temperatures warmed [27]. Consequently, we expect thermal sensitivity to climate modification since goats are presently restricted to northern climes typically at high elevation. Moreover, population growth in mountain goats is negatively associated with summer temperatures in an Alaskan study area [28,29]. Our efforts have concentrated in a realm where 87% of the local glaciers have disappeared in the last 100 years—Glacier National Park (GNP) in Montana—and bio-physical alterations have strikingly modified the ecosystem [30]. Understanding the relationship between warming temperatures relative to the melting of summer snow fields is useful to examine the potential for proximate thermal adjustments in presumptive cold-adapted mammals.

## Materials and methods

### Study site and subjects

Glacier National Park (48.6967° N, 113.7183° W) is a 4,100 km^2^ park, has between 1,885–3,269 mountain goats [31], and contains a full suite of native carnivores including those that prey on mountain goats; wolves (*Canis lupis*), mountain lions (*Puma concolor*), grizzly bears (*Ursus arctos*), black bears (*U*. *americanus*), and coyotes (*Canis latrans*) [26]. Data collection occurred from July to August (2013–2016) primarily at the Logan Pass and Sperry areas of GNP where habituated goats reside [32]. Both areas are subalpine-alpine environments and near the highpoint of mountains (2025 m elevation). Landcover is primarily rock, snow, conifer forest, and forb-dominated meadows. Because the goats are habituated, it was possible to record metrics on respiration (details below).

We avoided the possibility of pseudoreplication in our behavioral sampling by concentrating observations on 44 identifiable individuals, which were located on a near daily basis. Fourteen goats wore radio (ATS) collars, and eight carried satellite (Lotek Wireless) collars to allow for individual identification and to enable resource selection approximation. The satellite collars recorded locations every two hours. Due to the open nature of the alpine environment fix rates on collars were high (>90%), and therefore we removed outliers [33]. The other 20 goats had unique traits or temporary animal safe livestock paint to enable individual recognition. We also collected data on unmarked individuals but did not resample unidentifiable animals of the same age/sex class within an hour. All data collection occurred under an institutional animal care and use committee Animal Use Permit (017–15) from the University of Montana.

### Microhabitat choices—Resource selection models

Based on the literature review we developed a suite of abiotic and biotic covariates to determine mountain goat daily resource selection during the warmest part of any given summer day [29,34]. To accomplish this, we obtained and created remotely sensed landscape explanatory variables in a geographic information system (GIS) using ArcGIS 10.2 (ESRI, Redlands, California). We obtained an Advanced Spaceborne Thermal Emission and Reflection Radiometer (ASTER) 10 m digital elevation model (DEM) raster layer (earthexplorer.usgs.gov, accessed 10 March 2016). Using this DEM we produced an escape terrain layer which was the distance to areas with 60^o^ or higher slopes–we abbreviated this variable name to “60 slope distance” (Sarmento and Berger in review). Furthermore, we derived a four cardinal direction aspect layer from the DEM with the “flat” also being a category. We determined summer snow/ice extent using a remotely sensed vegetation map from August 1999 aerial photography. We validated the snow/ice layer using 2013 National Agriculture Imagery Program aerial imagery, which was the same year we initiated the study (https://www.fsa.usda.gov, accessed 9 September 2016). Although the snow/ice layers were created in 1999, the same locales retain snow and ice, whether glacial buildup or other year after year due to physical traits; depth and duration will vary due to insolation, wind, and temperature [35]. As a consequence, we relied on the earlier mapping exercise, and our field efforts provided ground-truth for snow/ice localities. Finally, we included a Moderate Resolution Imaging Spectroradiometer 17 class vegetation map to test for selection of landcover types (modis.gsfc.nasa.gov, accessed 10 March 2016).

To estimate mountain goat relative use of these environmental covariates we used a resource selection function (RSF). More specifically, we opted for a step selection function (SSF) with used and available points derived from location data [36–38]. We choose the SSF because it better accounts for fine scale differences in what is actually available to the mountain goats as each available paired unused location is conditioned on where the goat had been. Locations for eight satellite collars equated to used pixels, while we randomly generated available locations based on a distribution of turning angles and step lengths for each individual goat, which equates to third order habitat selection, i.e. where an animal chooses to go within its home range [39]. Two adult males wore satellite collars, and six were affixed to adult females. We subsetted locations to July-August 2014–16 and to the hottest periods during the day (12:00–18:00) when we expected heat-mediated habitat selection most likely. Available locations were derived from mountain goat turning angles and step lengths which were calculated from the location data using the movement pathometerics function in the program Geospatial Modeling Environment (GME) version 0.7.2 [40]. Five paired available locations were then randomly created for each location point based on the turning angle and step length distributions using the movement.ssfsamples function in GME. We chose five paired case-control locations to minimize contamination. Both available and used locations were then intersected with habitat covariates using GME. Nevertheless, limitations for developing inferences are associated with matrices based on use-availability data; predicted values are an exponential approximation to logistic regression and resource-selection probabilities are relative values that are not scaled between 0 and 1. RSFs, however, are directly related to actual probability of resource use [41].

To examine resource selection (use versus available) in mountain goats, we used a conditional multiple logistic regression (match case-control) in the program R version 3.3.1 [42]. We also examined weather variables that could be correlated with goat distance to snow during summer afternoons using univariate linear models because of the possibility that snow use occurs either as a thermal or insect refuge. We obtained data on temperature, wind, humidity, and solar radiation from a weather station located at the Logan Pass study site. For our distance-to-snow linear model we only tested the univariate influence of covariates since weather variables are highly correlated (>80%). For our SSF we developed a global model with all explanatory variables and then removed nonsignificant variables (P > 0.1) until only significant variables remained, while simultaneously monitoring coefficients, log-likelihood, and significances for large change (> 20%) during each removal [43,44]. We started with this backward stepwise model selection method to objectively eliminate variables that were potentially not important in explaining mountain goat habitat selection. We tested independence of covariates via 1) a variance inflation factor of less than five, and 2) whether correlation coefficients were under 40% between parameters [44].Further, we tested how our each of our backward stepwise models ranked compared to 1) univariate models, and 2) models without distance to snow so we also employed small size correction for Akaike Information Criterion (AICc) for model ranking [45]. We choose this approach because we were interested how much influence each variable had alone, as well as how distance to snow influenced model rank. Therefore, our top model held the highest AICc weight–which describes the relative likelihood of that model relative to the competing models [46]. We present results as an odds ratio—which is derived by exponentiating the beta coefficient–because the interpretation is more biologically understandable. Use of conditional logistic regression models prohibits the use of area under the curve and k-folds estimates as these diagnostic tests are not currently available for this relatively new resource selection statistic [38].

### Estimating microclimate effects and respiration

From July-August 2013–16 we quantified mountain goat time budgets during 180 second focal bouts on both identifiable and unidentifiable individuals. Among the abiotic variables we recorded were; cloud cover, wind, and temperature, the latter two with a Kestrel 2000 wind and weather meter. Cloud cover was assessed by partitioning the sky into quadrants and estimating the percentage of cloud cover within each section. The shading of the goat was classified as an individual in; fog, overcast, dark, sun, or shade. Location and linear distances to escape terrain, snow, and to observer were estimated by a Bushnell rangefinder or with topographic map in a Garmin E-Trex Vista Global Positioning System (GPS). We defined escape terrain as rock cliffs with slopes of 60^0^ or steeper (Sarmento and Berger in review). Land cover was classified categorically where the focal sample ended (classes included: snow, cliff, meadow, forest, and scree). The sex and age of goats were established by examining group structure, horn/ body morphology, urination postures. We counted goat breaths per minute during focal samples by using a hand counter and stopwatch while examining an individual’s chest or mouth movement to determine air inhalation. We excluded one respiration datapoint, which was eight breaths per a minute, which was an outlier and apparently a miscalculation.

We also accounted for inter-observer reliability in our analyses because different staff were involved in data recording. A categorical variable for observer was included in models to account for differences between data recorders. Behaviors were represented by the activity with the highest proportion of time within the180 second bouts, and included: vigilance, bedded, moving, feeding, agonism, and grooming. Vigilance is defined as an individual with its head at, or above, the shoulders and not moving [47,48]. Feeding involved the act of removing vegetation; movement was locomotion with an animal’s head at or above its shoulders. We also quantified the percentage of winter coat remaining on individuals because heat load is suspected to affect breath rate; the % of remaining winter coat was categorized as 0, 1–25, 26–50, 51–75, and 76–100.

The influence of abiotic and biotic factors on mountain goat respiration was estimated using quasi-Poisson generalized linear model in program R version 3.3.1 [42]. We choose a quasi-Poisson model to account for overdispersion of the respiration count data. The “quasi” state allows the variance to be a linear function of the mean as opposed to the assumption that the variance equals the expected value, which is the case with the straight Poisson models. Distances to snow were not normally distributed with a high number of true zeros and therefore we categorized distances as goats on snow, near to (≤ 20 m), or away from snow (≥20 m). To facilitate analyses, we categorized activities during the observation and retained the behavior with highest proportional time. We used backwards stepwise model selection to obtain a top quasi-Poisson generalized linear model explaining mountain goat respiration, although there are some considerations when using this method [49]. We choose a threshold P-value of .01 to systemically drop the least significant variables until only significant variables remained. Quasi-Poisson models do not have a likelihood and therefore do not produce AIC values for model ranking. Thus our top model is the most parsimonious and only contains significant covariates.

We quantified differences in surface temperature across microclimatic sites using data loggers, which recorded temperature on an hourly basis (Onset Hobo Logger, Bourne Massachusetts). Two data loggers were placed in each of four different locations: forest shade, open meadow, rocks, and snow. We controlled for elevation and aspect in our placement of temperature loggers. To analyze temperature data, we subsetted, as indicated above, by the warmest period of the day (12:00–18:00). We tested for differences in temperature between microclimatic sites using a Welch two sample t-test that adjusts for unequal variance. Alpha level was set a priori at 0.01.

## Results

### General patterns of resource selection

We obtained 5,251 satellite fixes during July-August 2014–2016, which were matched to 26,255 available locations. Compared to four other weather measures, temperature best explained mountain goat distance to snow on summer afternoons by carrying 90% of AICc weights (Table 2). Including the second model, solar radiation, and the top models carried 100% of AICc weights. While the modelled distance to snow shows a biologically trivial, but statistically significant, increase in goat proximity to snow (β = -0.05, S.E. = 0.01) for each one-degree C increase in temperature. Goats were 17.46 meters (S.E. = 0.70) from snow, on average, during hot summer afternoons when accounting for temperature.

**Table 2:** Competing weather models explaining mountain goat distance to snow. Model selection of univariate weather variables associated with mountain goat distance to snow during summer afternoons (12:00–18:00); small size corrected by Akaike Information Criterion. Data are from eight GPS collared mountain goats in Glacier National Park from 2013–2016.

Variable	Δ AICc	AICcWt	Log Likelihood
temperature	0.00	0.90	-13205.16
solar radiation	4.50	0.10	-13207.42
humidity	41.67	0.00	-13226.00
wind speed	57.16	0.00	-13233.74
wind gust speed	60.74	0.00	-13235.54

**Fig 1:**
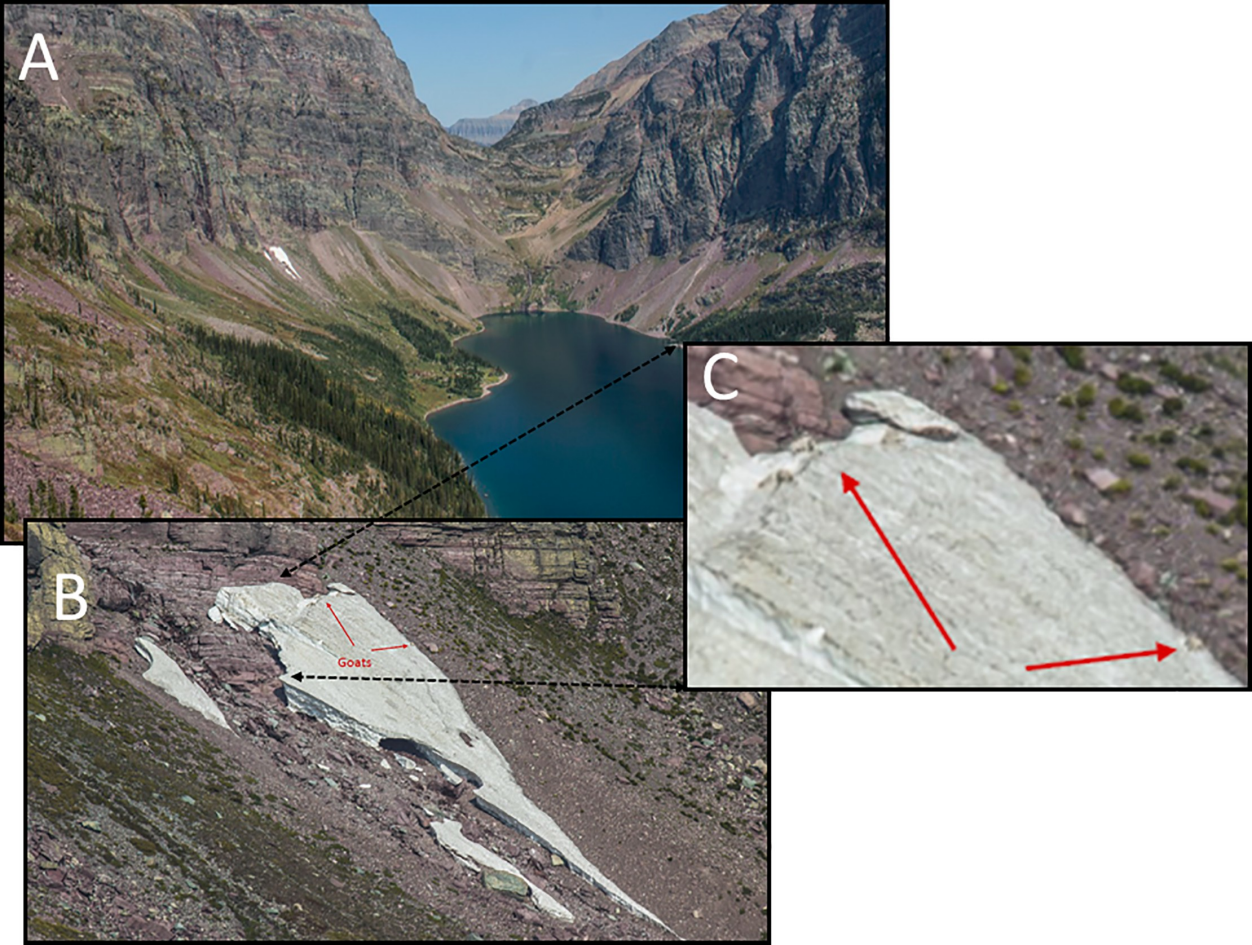
Example of mountain goat selection for snow on a hot summer afternoon.

**Fig 2:**
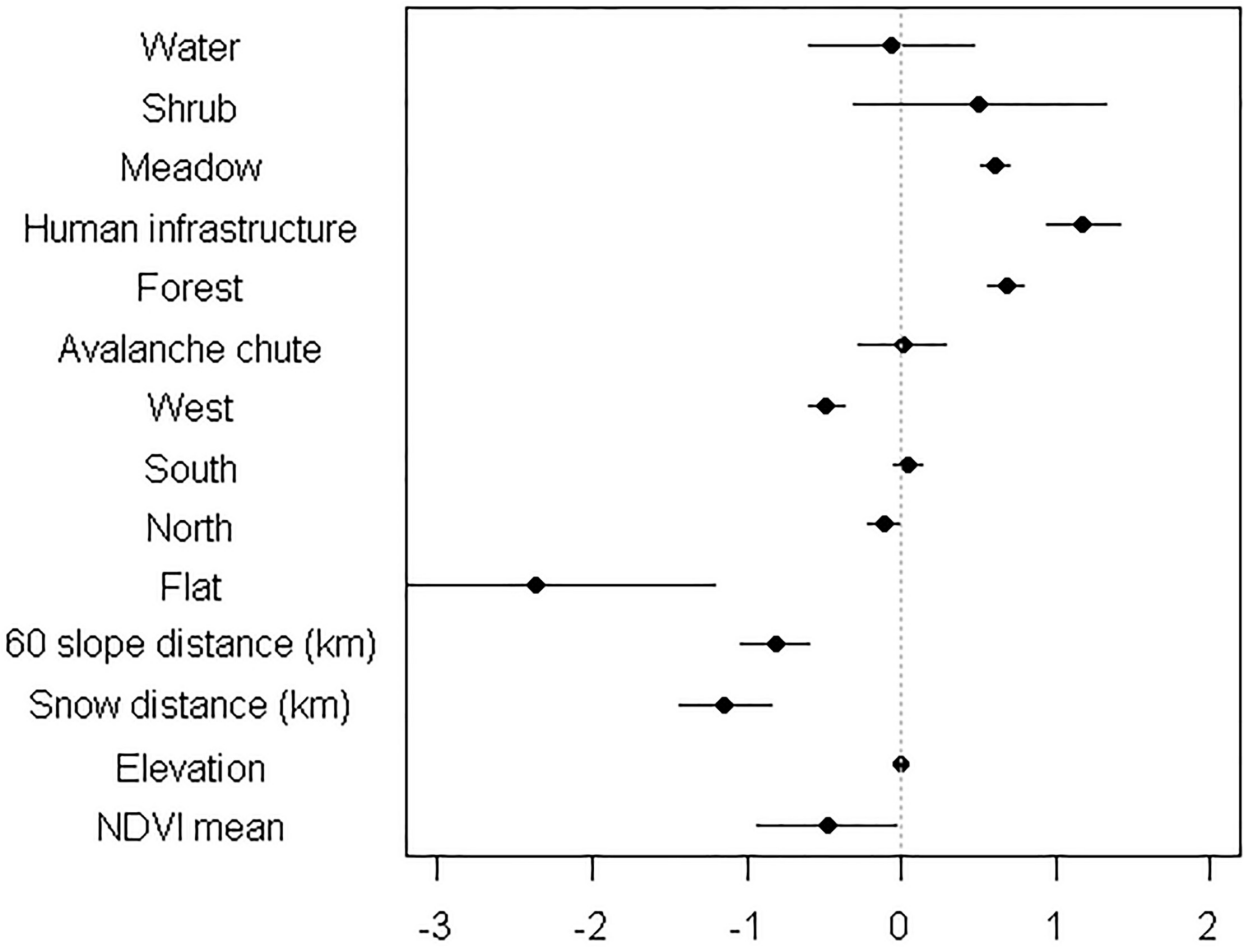
Coefficient estimates for mountain goat resource selection. Coefficient estimates from the top match-case control multiple logistic regression model of mountain goat resource selection during July and August afternoons (12:00–18:00). Data are from eight GPS collared adult mountain goats in Glacier National Park from 2014–2016. Baseline for aspect is east and for landcover is rock. Error bars represent 95% confidence intervals. Elevation had a S.E. of <0.00. The variable 60 slope distance is how close mountain goats were to escape terrain (60° slopes).

Relative to the physical landscape, mountain goats displayed strong selection for areas closer to escape terrain and snow (Figs 1 and 2). For every 1 km increase in distance from snow the odds of goat use decreased 68% (S1 Table). Conversely, for every 1 km increase in distance from slopes greater than 60° goat use decrease 56%. The odds of mountain goat using a specific location increased 98% when the landcover was forest, and 226% for landscapes lacking in human infrastructure. Additionally, mountain goat use increased 84% for meadow cover and 5% for southern aspects. Goats avoided flat and western aspects (99% and 48% reduction in odds of use, respectively). The top two models explaining goat resource selection included 99% of model weights (S2 Table).

### Microclimate effects on respiration

**Fig 3:**
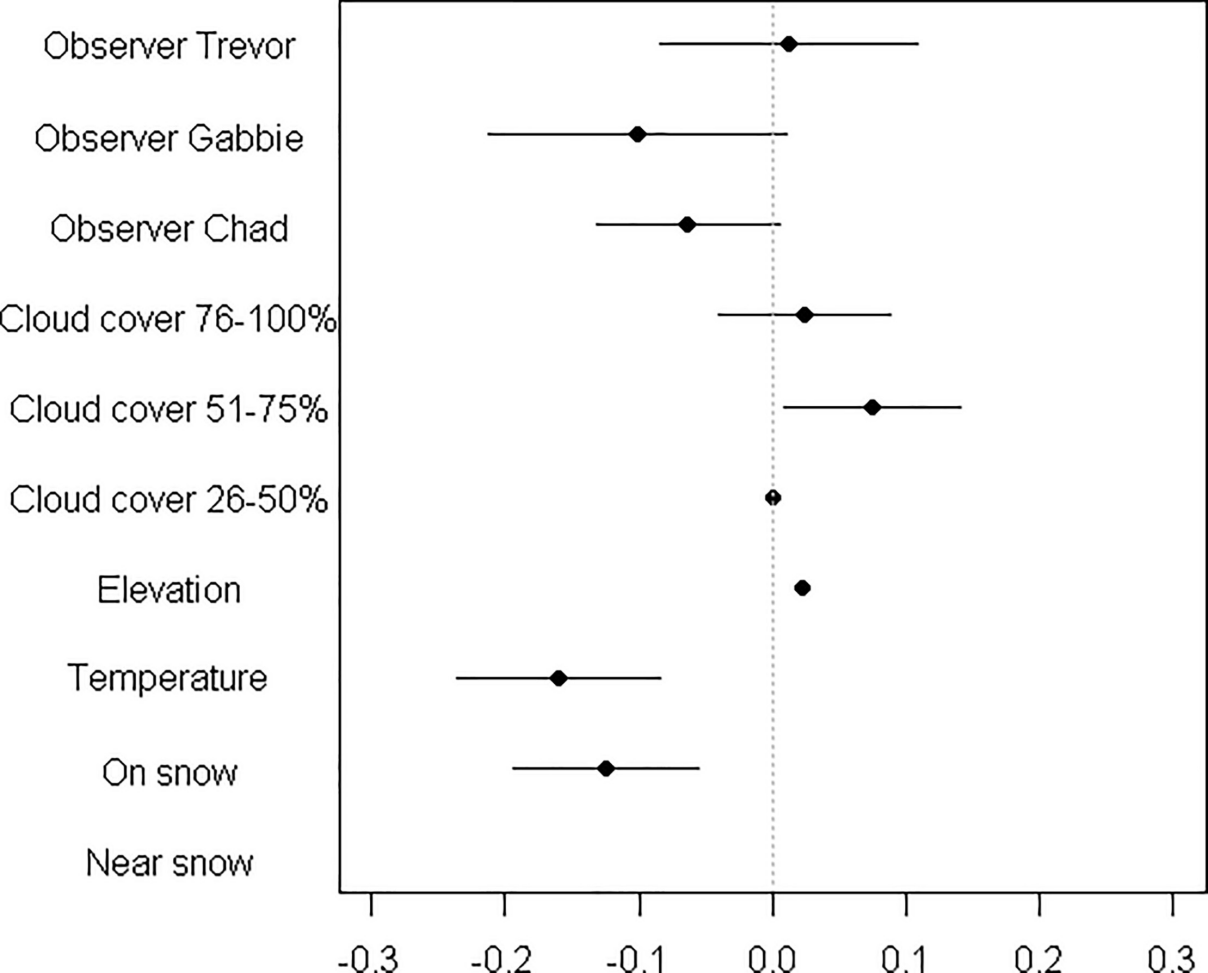
Coefficient estimates for variables influences mountain goat respiration. Coefficient estimates from the top linear model explaining mountain goat breaths per minute in Glacier National Park 2014–2016. Baseline values include the primary author as an observer constant, 0–25% cloud cover, and away from snow. Error bars represent 95% confidence intervals. Variation was small for temperature and elevation with a standard errors of less than 0.01 each. We accounted for observer variability by including each field technician; Trevor, Chad, and Gabbie. Data were from 44 identifiable individuals and unmarked goats. “Near snow” was defined as a goat less than 20 meters from a snow patch.

**Fig 4:**
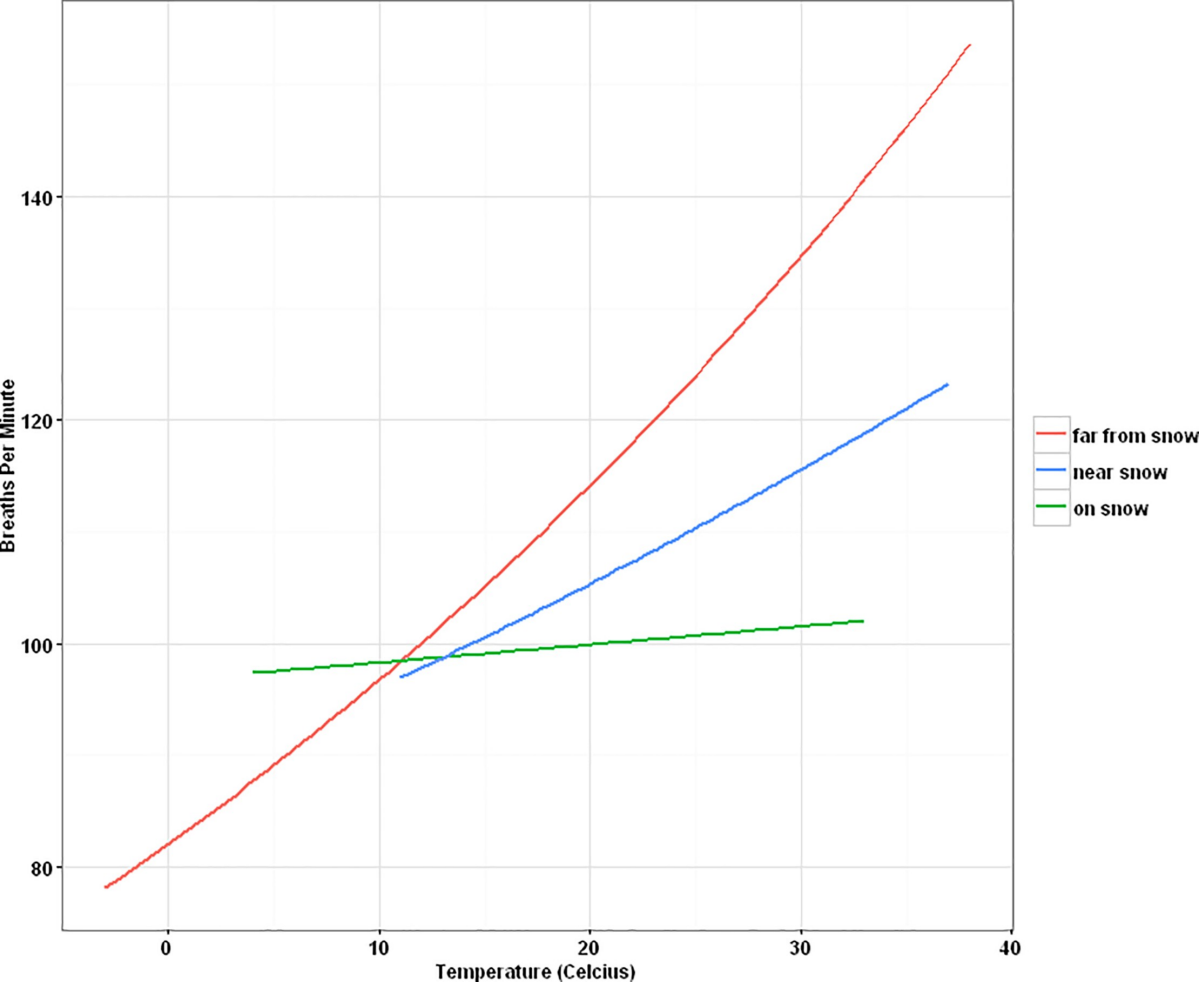
Mountain goat respiration in response to temperature and distance to snow. Estimated mountain goat respiration based as a function of temperature and distance to snow based on top quasi-Poisson model. “Near snow” was defined as a goat less than 20 meters from a snow patch.

We collected a total of 473 observations on mountain goat respiration (mean = 108.38 breaths per minute (BPM) ± 1.50 S.E.) from identifiable and unidentifiable individuals (BPM range from 19–195). Seven variables were dropped from the top respiration quasi-Poisson model for being non-significant including; distance to people, distance of separation, percentage of winter coat remaining, shading, wind, behavior, and sex/age of the animal. Goat proximity to snow affected respiration, with breathing rate declining on average 15% on snow (Fig 3 and S3 Table). Goats near snow (≤ 20 m) had a 11.5% reduction in respiration. Elevation also influenced breathing significantly—goat respiration increased by 1% for every 100-meter increase in altitude. Each one-degree Celsius ambient air temperature increase led to a 1% increase in BPM (Fig 4). Cloud cover also influenced goat respiration with less clouds leading to more breathing. Observers overlapped with the primary author (WS) in quantifying goat breaths except one biotech, who consistently recorded higher respirations (22% BPM more on average). Other factors did not influence mountain goat respiration significantly. Finally, we inferred heat stress in 104 occurrences, whereupon individuals panted with mouths open, and three times with tongues exposed while panting–we still recorded breathing rates in these situations.

With respect to microhabitat variation in surface temperature, rocky landcover averaged 14.53° C (± 0.17 S.E.) and meadows 14.27° C (± 0.11 S.E.)–with no difference between these cover types (T = 1.26, DF = 9980.24, P = 0.21). Data loggers on snow recorded an average temperature of 9.95° C (± 0.11 S.E.), and in the forest shade temperature was 10.61° C (± 0.54 S.E.), with snow being significantly colder than shade (T = -5.59, DF = 14358.63, P = <0.01). Both shade and snow were significantly cooler than meadows (T = -30.14, DF = 19624.42, P = <0.01; T = -28.58, DF = 22316, P = <0.01, respectively). The Logan pass weather station mean temperature from 12:00–18:00 during July and August 2014–16 was 13.66 C° C (± 0.17 S.E.), while average wind speed was 13.78 kph (± 0.17 S.E.).

## Discussion

We developed three predictions that stem from the more general hypothesis that snow patch use benefits mountain goats through a more favorable thermal environment. For the first–that individuals associate with snow patches in a non-random fashion–the evidence was strong (Figs 1 and 2). Mountain goats selected persistent summer snow patches, a behavior which serves to enhance thermoregulatory abilities by reducing metabolic costs through lower respiratory rates. In turn, our empirical findings and associated analyses also supported the second prediction that resting on snow patches reduced respiration. Goats also occurred more frequently closer to snow than at random point locations during warm summer afternoons, and when resting on snow breathing rates decreased by about 15%. With respect to the test of our third prediction–that snow patches receive relatively higher usage than ridges on windy days–we fell short. We could not adequately test the proposition due to a sample size limitation and because we did not measure insect densities directly. As a consequence, we proffer a qualitative argument that use of snow is consistent with thermoregulatory benefit and not directly an inevitable consequence of insect avoidance. But both factors are likely in play. Nevertheless, if habitat selection for snow was primarily a response to insects, we’d have expected wind to be the primary determinant of goat snow use. Putative insect harassment on hot summer afternoons is most influenced by wind speed [50,51], while mountain goat selection for snow was markedly less influenced by winds (Table 2).

Relationships between thermoregulatory benefit and parasitizing insects are complex and affected not only by temperature and wind, but also social grouping, available habitat, and type of pestering insect. Caribou, for example, select windier locations during severe insect agitation [50]. At our study area, the primary biting insects–mosquitoes (*Culicidae*) and black flies (*Simuliidae*)–would also be highly affected by wind speed and our Logan Pass realm was consistently windy with low levels of variability (13.78 kph ± 0.17 S.E.). While snow patches were small during our period of study (<300 m), we expected them not to have reduced above surface air temperatures sufficient enough to influence insect abundance, a hypothesis in need of testing. However, we noted insect harassment of goats to be slight at least as judged by individuals with only moderate flicking of ears and head both on and off snow. If insect harassment was driving mountain goat habitat selection, we would have expected 1) individuals to select for windswept ridges devoid of snow because wind has the largest effect on insect activity, 2) that winds would drive goat snow use because insects are most influenced by wind speed, and 3) that Logan Pass would have low winds that would facilitate insect activity. Hence, our belief is that goats did not use snow to escape insects per se, as we frequently observed heat stressed goats move to snow.

A fuller understanding of how temperature specifically affected goat movements to snow was undoubtedly hampered by our small sample size (N = 8) of GPS collared goats despite other aspects of our findings being based on 44 individuals. For instance, and with respect to the former, for each degree C^o^ of ambient increase above baseline individuals were only 0.05 m closer to snow. Such trivial movement as understood in our model is likely a product of goats already residing close to snow on hot summer afternoons (17.46 meters away on average). Selection for snow was so influential that the odds of mountain goat use of an area decreased 68% for every 1 km further from snow patches–which was greater than their famous selection for escape terrain. Goats clearly show affinity of snow for thermoregulatory benefits on warm days including respiration is clear (Figs 1 and 2). An uneasiness between statistical constraint and biological inference is clearly reflected by the logistics of small samples in other difficult-to-study cold adapted mammals. For example Mongolian gazelles are suspected of avoiding railways, an extrapolation based on only two collared animals in a population in excess of a third of a million [52]. Or, explicitly in power analyses such as the case of estimating adult survival in bison; more than 700 animals in a population would be needed to avoid a Type II error when total population sizes are far below this statistically ideal number [53].

With respect to goats, and other species of the Rocky Mountains and boreal North America, as climate warms negative impacts are likely to increase at least along boundaries with the greatest increases in temperature. Beyond broad scale alterations of geographical ranges, behavioral adjustments may be detectable. Moose (*Alces alces*) increase their respiration in association with rising temperature [54] and also mediate body temperature by selecting shade of thick forests in summer heat [55,56]. The extent to which moose use snow in relation to heat remains unclear although increasing temperature negatively affect the quality of their primary forage [57] and plant phenological development [58]. We know little of longer-term impacts except that species like muskoxen or pikas (*Ochotona princeps*) have disappeared from the southern portions of their range, sites once steeped at peri-glacial margins or other colder climes that have now warmed [59]. And, we lack knowledge about whether current behavioral tactics to minimize heat stress will promote longer term viability.

Some more proximate trends are becoming clear across short time frames though. Mountain goat population growth in parts of Alaska is negatively correlated with summer temperatures [28,29], although an understanding mechanisms why this is occurring remains elusive. Whether summer snow is requisite is arguable from different directions. Mountain goats were introduced to the Black Hills of South Dakoda in 1924, and still persist in healthy numbers despite the absence of summer snowpack. On the other hand, introduced populations are more robust than native ones in population growth, perhaps because those habitats are less prone to density-dependence across short time frames [60,61]. Hence, it’s unclear the extent to which snow on native ranges offers demographic, rather than immediate thermal, benefits to goats. Climate signals must exist because species like Harrington’s mountain goat, which inhabited the Grand Canyon and became extinct 11,190 years ago, as did more southerly populations of other species [27,59].

Interactions between available habitat and population size are of course complex in predicting persistence because rarely is causation singularly explained and many mechanisms may be operating simultaneously [62]. In Alaska’s coastal mountains, summer habitat for goats is predicted to shrink up to 86% over the next 70 years [29,63]. Forest encroachment, there, as in Glacier National Park where conifers are moving upslope [30], will affect goats in three principal ways: fragmentation of habitat, decreased food availability [64], and reduced predator detection [26].

Cold-adapted mammals of high elevation share commonalities in tactics to maintain homeostasis but also can employ quite different behavioral means (Table 1). Climate challenge will affect taxa differently by season and through stressors that vary abiotically and biotically [1,65], often directly through body condition because warmer or colder winters affect fecundity, neonate size, and sexual maturation [66]. We know that climate change will be a challenge, but we know less about the actual mechanisms, especially for little known species such as mountain goats.

## Supporting information

S1 Table Mountain goat resource selection coefficient estimates. Coefficient estimates from the top match-case control multiple logistic regression model of mountain goat resource selection during July and August afternoons (12:00–18:00). Data are from eight GPS collared mountain goats in Glacier National Park from 2014–2016. Baseline for aspect is east and for landcover is rock.
(DOCX)

S2 Table Mountain goat resource selection competing models. Model selection of mountain goat resource use during July and August afternoons (12:00–18:00). Data are from eight GPS collared mountain goats in Glacier National Park from 2014–2016.
(DOCX)

S3 Table Coefficient estimates for mountain goat respiration model. Coefficient estimates from the top linear model explaining mountain goat breaths per minute in Glacier National Park 2014–2016. Baseline values include the primary author as an observer constant, 0–25% cloud cover, 0% winter coat, and away from snow.
(DOCX)

S1 Data Mountain goat behavior data. Observation data collection on mountain goats in Glacier National Park 2014–2016. DOS stands for distance of separation between subject and observer. BPM stands for breaths per minute.
(CSV)

S2 Data Mountain goat collar data. Satellite collar data from mountain goats with covariates intersected for resource selection analyses. NDVI stands for Normalized Difference Vegetation Index. Elev stands for elevation. Snowdis stands for distance to snow. Slope60 stands for distance to 60 degree slopes.
(CSV)
