# Correction: Seeking snow and breathing hard – Behavioral tactics in high elevation mammals to combat warming temperatures

**DOI:** 10.1371/journal.pone.0228820

**Published:** 2020-01-30

**Authors:** Wesley Sarmento, Mark Biel, Joel Berger

In Fig 3, the coefficient estimates are misaligned to the variable names. As a result, one the coefficient estimates have been omitted. The authors have provided a corrected Fig 3 here.

**Fig 3:**
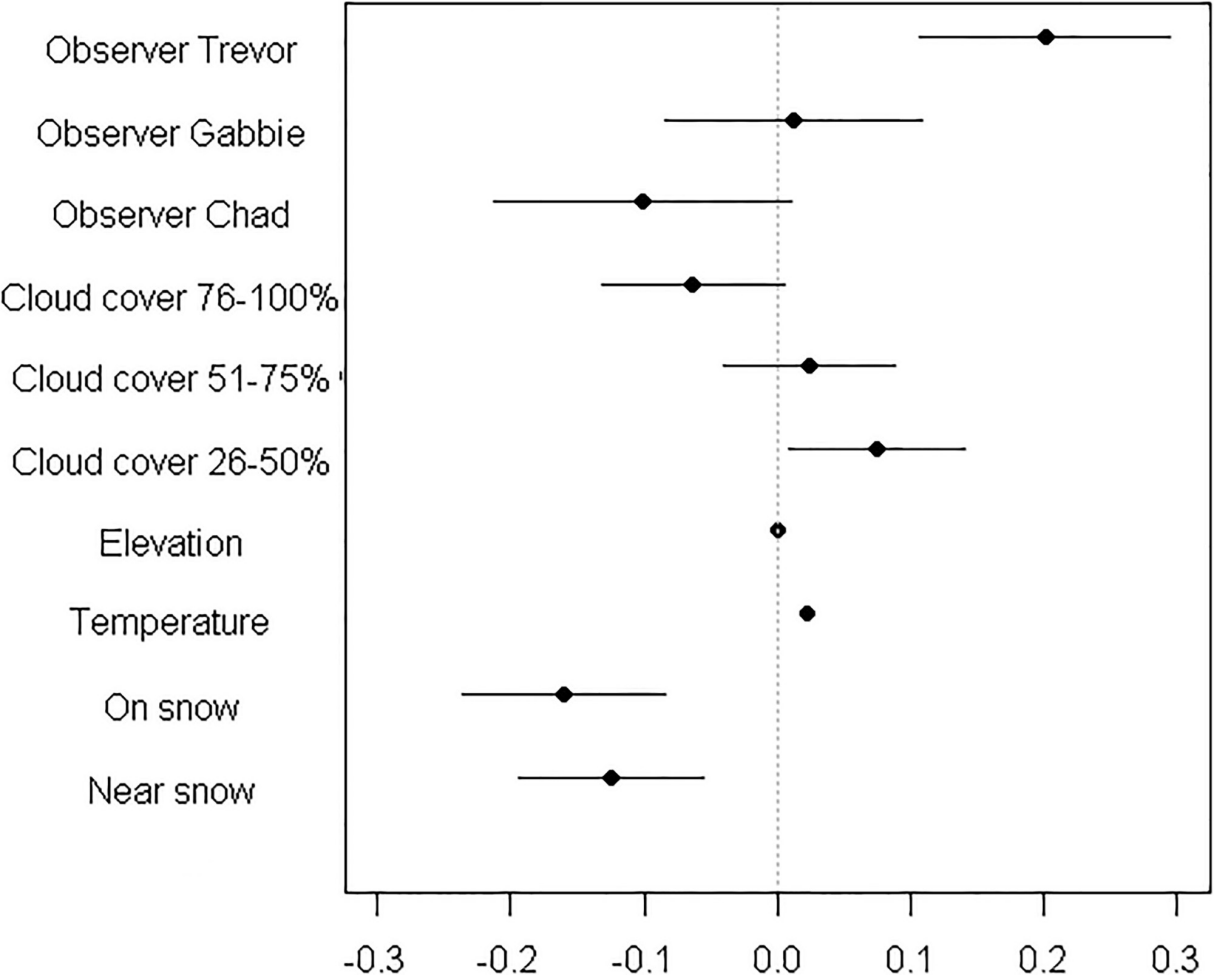
Coefficient estimates for variables influences mountain goat respiration. Coefficient estimates from the top linear model explaining mountain goat breaths per minute in Glacier National Park 2014–2016. Baseline values include the primary author as an observer constant, 0–25% cloud cover, and away from snow. Error bars represent 95% confidence intervals. Variation was small for temperature and elevation with a standard errors of less than 0.01 each. We accounted for observer variability by including each field technician; Trevor, Chad, and Gabbie. Data were from 44 identifiable individuals and unmarked goats. “Near snow” was defined as a goat less than 20 meters from a snow patch.
