# Supplementary material for: Seeking snow and breathing hard – Behavioral tactics in high elevation mammals to combat warming temperatures
Source: PLoS One. 2019 Dec 11;14(12):e0225456. doi: 10.1371/journal.pone.0225456 (PMC6905581; doi:10.1371/journal.pone.0225456)
Supplement: S1 Table — Coefficient estimates from the top match-case control multiple logistic regression model of mountain goat resource selection during July and August afternoons (12:00–18:00). Data are from eight GPS collared mountain goats in Glacier National Park from 2014–2016. Baseline for aspect is east and for landcover is rock. (DOCX) [file pone.0225456.s001.docx]

**S1 Table.**

| Variable | β | exp(β) | S.E. | Z score |
| --- | --- | --- | --- | --- |
| NDVI mean | -0.48 | 0.62 | 0.23 | -2.06 |
| Elevation | 0.00 | 1.00 | 0.00 | 8.14 |
| Snow distance (km) | -1.14 | 0.32 | 0.15 | -7.36 |
| 60 slopes (km) | -0.81 | 0.44 | 0.11 | -7.19 |
| Flat | -2.37 | 0.09 | 0.60 | -3.99 |
| North | -0.11 | 0.89 | 0.05 | -2.35 |
| South | 0.05 | 1.05 | 0.05 | 0.99 |
| West | -0.49 | 0.62 | 0.06 | -8.06 |
| Avalanche chute | 0.01 | 1.01 | 0.14 | 0.08 |
| Forest | 0.68 | 1.98 | 0.06 | 11.85 |
| Human infrastructure | 1.18 | 3.26 | 0.12 | 9.49 |
| Meadow | 0.61 | 1.84 | 0.05 | 12.34 |
| Shrub | 0.51 | 1.66 | 0.42 | 1.23 |
| Water | -0.06 | 0.94 | 0.27 | -0.23 |
