# Supplementary material for: Seeking snow and breathing hard – Behavioral tactics in high elevation mammals to combat warming temperatures
Source: PLoS One. 2019 Dec 11;14(12):e0225456. doi: 10.1371/journal.pone.0225456 (PMC6905581; doi:10.1371/journal.pone.0225456)
Supplement: S2 Table — Model selection of mountain goat resource use during July and August afternoons (12:00–18:00). Data are from eight GPS collared mountain goats in Glacier National Park from 2014–2016. (DOCX) [file pone.0225456.s002.docx]

**S2 Table**

| Model | K | Δ AICc | AICcWt | LL |
| --- | --- | --- | --- | --- |
| NDVI + elevation + snowdistance + 60 slopes + aspect + cover | 14 | 0.00 | 0.76 | -8980.07 |
| elevation + snowdistance + 60 slopes + aspect + cover | 13 | 2.26 | 0.24 | -8982.20 |
| elevation + snowdistance + aspect + cover | 12 | 53.35 | 0.00 | -9008.75 |
| elevation + aspect + cover | 11 | 122.39 | 0.00 | -9044.27 |
| elevation + cover | 7 | 272.39 | 0.00 | -9123.27 |
| elevation + cover | 7 | 272.39 | 0.00 | -9123.27 |
| Elevation | 1 | 506.99 | 0.00 | -9246.57 |
|  |  |  |  |  |
