# Supplementary material for: Seeking snow and breathing hard – Behavioral tactics in high elevation mammals to combat warming temperatures
Source: PLoS One. 2019 Dec 11;14(12):e0225456. doi: 10.1371/journal.pone.0225456 (PMC6905581; doi:10.1371/journal.pone.0225456)
Supplement: S3 Table — Coefficient estimates from the top linear model explaining mountain goat breaths per minute in Glacier National Park 2014–2016. Baseline values include the primary author as an observer constant, 0–25% cloud cover, 0% winter coat, and away from snow. (DOCX) [file pone.0225456.s003.docx]

**S3 Table.**

| Variable | β | Exp(β) | SE | T-Value |
| --- | --- | --- | --- | --- |
| (Intercept) | 3.27 | 26.36 | 0.19 | 16.99 |
| Near snow | -0.12 | 0.88 | 0.04 | -3.56 |
| On snow | -0.16 | 0.85 | 0.04 | -4.10 |
| Temperature | 0.02 | 1.02 | 0.00 | 9.72 |
| Elevation | 0.00 | 1.00 | 0.00 | 5.33 |
| Cloudgroups26-50 | 0.07 | 1.08 | 0.03 | 2.24 |
| Cloudgroups51-75 | 0.02 | 1.02 | 0.03 | 0.75 |
| Cloudgroups76-100 | -0.06 | 0.94 | 0.03 | -1.82 |
| Observer Chad | -0.10 | 0.90 | 0.06 | -1.79 |
| Observer Gabbie | 0.01 | 1.01 | 0.05 | 0.25 |
| Observer Trevor | 0.20 | 1.22 | 0.05 | 4.17 |
